# SLC25A39 Upregulation Is Associated with DNA Methylation, Immune Cell Infiltration, and Poor Prognosis in Hepatocellular Carcinoma

**DOI:** 10.3390/ijms27073098

**Published:** 2026-03-28

**Authors:** Yifei Mo, Zhipeng Du, Mei Liu

**Affiliations:** 1 Department of Gastroenterology, Institute of Liver and Gastrointestinal Diseases, Tongji Hospital, Tongji Medical College, Huazhong University of Science and Technology, Wuhan 430030, China; moyifei@hust.edu.cn; 2 Grade 2022 of Paediatrics, Second Clinical College, Tongji Hospital, Tongji Medical College, Huazhong University of Science and Technology, Wuhan 430030, China

**Keywords:** SLC25A39, hepatocellular carcinoma, DNA methylation, tumor immune microenvironment, prognosis, biomarker

## Abstract

Solute carrier family 25 member 39 (SLC25A39) is a pivotal mitochondrial glutathione transporter and an emerging oncoprotein in hepatocellular carcinoma (HCC). While its cell-intrinsic roles are increasingly recognized, its comprehensive functions in modulating the tumor immune microenvironment (TIME) and epigenetic landscape within HCC remain undefined. To address this, we employed an integrated multi-omics and experimental approach, including TCGA, ssGSEA, CCK-8, Transwell, etc. Our study confirmed SLC25A39 upregulation and its pro-tumorigenic role. Notably, we provide several key novel insights: First, we establish the first link between *SLC25A39* promoter hypermethylation at specific CpG sites and poor patient prognosis, revealing an epigenetic regulatory layer in HCC. Second and most importantly, we pioneer the exploration of SLC25A39 in the HCC immune context, demonstrating its association with a distinct immunosuppressive TIME characterized by a Th2-skewed profile, reduced cytotoxic cell infiltration, and elevated immune checkpoint (CTLA-4, PD-1) expression. Furthermore, drug sensitivity analysis linked SLC25A39 to a broader spectrum of pharmacological agents beyond sorafenib. Collectively, our findings not only reinforce SLC25A39 as a therapeutic target but, for the first time, reposition it as a potential modulator at the intersection of tumor metabolism, epigenetics, and immunology in HCC, offering a rationale for its inhibition, particularly combined with immunotherapy.

## 1. Introduction

Globally, in 2022, liver cancer accounted for an estimated 865,000 new cases and 757,948 deaths, causing it to be the third most lethal cancer (after lung and colorectal) and the sixth most common in terms of diagnosis [1]. Projections indicate a concerning rise, with the annual mortality figure expected to exceed 1.3 million by 2040 [2]. Primary liver cancer consists mainly of hepatocellular carcinoma (HCC) (75–85%), followed by intrahepatic cholangiocarcinoma, which constitutes 10–15% of cases [1]. Despite rapid advancements in HCC management over the past decade, including innovations in surgery and targeted therapy, the overall survival rate for patients remains poor [3]. Consequently, there is a pressing need to deepen the understanding of HCC’s pathogenic mechanisms to facilitate the development of innovative treatment strategies.

Mitochondrial dysfunction, a hallmark of cancer, contributes to oxidative stress and metabolic reprogramming, facilitating tumor growth and metastasis in HCC [4]. Members of the solute carrier family 25 (SLC25), which regulate mitochondrial metabolite transport, are increasingly implicated in HCC [5,6,7]. Among them is SLC25A39, a critical mitochondrial glutathione (GSH) transporter essential for redox and iron homeostasis [8,9]. GSH is the major antioxidant that neutralizes reactive oxygen species (ROS) generated during oxidative phosphorylation. By maintaining the mitochondrial redox balance, SLC25A39 protects against oxidative damage to mitochondrial Deoxyribonucleic Acid (DNA), proteins, and lipids, thereby ensuring proper metabolic function and preventing the initiation of apoptosis [8]. SLC25A39 is consistently overexpressed in HCC tissues and drives tumor cell proliferation, invasion, and therapy resistance (e.g., to sorafenib) [10,11,12,13]. Mechanistically, these effects are mediated through its fundamental role in mitochondrial physiology. By ensuring adequate mitochondrial GSH, SLC25A39 modulates key signaling pathways (e.g., Hedgehog), fine-tunes mitochondrial ROS signaling, and, critically, enables cancer cells to evade ROS-induced cell death pathways such as ferroptosis and necroptosis [10,11,12,13]. Thus, SLC25A39 functions as a linchpin in the mitochondrial adaptation of HCC cells, supporting their aggressive phenotypes.

The tumor immune microenvironment (TIME) is a complex ecosystem that consists of immune cells (e.g., CD8^+^ T cells), stromal cells (e.g., cancer-associated fibroblasts), extracellular matrix (e.g., fibronectin) and soluble signaling molecules (e.g., interleukin-6), and this environment enables tumor cells to evade immune surveillance, sustain proliferation, and drive metastasis in HCC [14]. As the major intracellular antioxidant, GSH plays a crucial role in regulating the tumor immune microenvironment [15] and acts as a regulator of antitumor immunity and the TIME [16]. In colorectal cancer, SLC25A39 upregulation is linked to an immunosuppressive phenotype [17]. However, two critical aspects of SLC25A39’s function in HCC remain largely unexplored: (1) its role in shaping the local immune landscape, and (2) its regulation by epigenetic mechanisms such as DNA methylation and associated prognostic implications.

Therefore, this study aimed to investigate these unexplored dimensions of SLC25A39 in HCC. We confirmed its upregulation and prognostic significance. Importantly, we provide the first comprehensive analysis linking SLC25A39 to DNA methylation and its prognostic implications in HCC. Furthermore, we uncovered the association between SLC25A39 and a distinct immunosuppressive TIME, characterized by altered immune cell infiltration (T helper 2-skewed profile) and elevated checkpoint molecule expression (Cytotoxic T-Lymphocyte Associated Protein 4, CTLA-4; Programmed Cell Death protein 1, PD-1). Additionally, drug sensitivity analysis indicated the correlation between SLC25A39 expression and drug sensitivity profiles beyond sorafenib. Finally, in vitro experiments using distinct HCC cell lines validated its role in facilitating malignant behaviors. Collectively, our study solidifies SLC25A39 as an oncoprotein and reveals its novel role as a multimodal regulator operating across metabolic, epigenetic, and immune axes in HCC. This underscores its translational promise and specifically motivates the exploration of novel combinatorial strategies with immunotherapies.

## 2. Results

### 2.1. The Expression of SLC25A39 in Pan-Cancer and HCC

**Figure 1:**
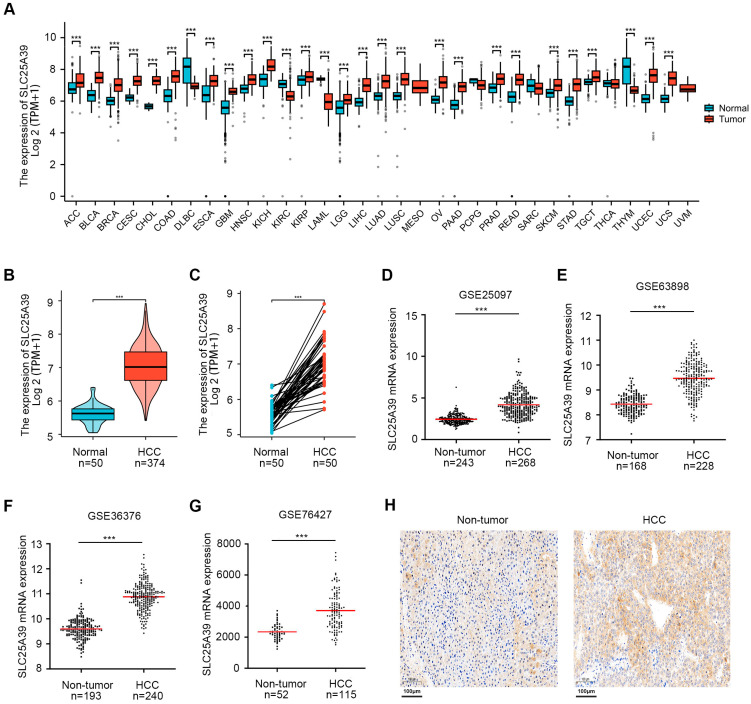
**The expression of SLC25A39 in pan-cancer and HCC.** (**A**). Pan-cancer analysis of *SLC25A39* mRNA expression across tumor and adjacent normal tissues from the XENA TCGA GTEx-ALL database, demonstrating frequent upregulation in multiple cancer types. (**B**,**C**). *SLC25A39* mRNA expression is significantly elevated in HCC tumor tissues compared to normal liver tissues in the TCGA-LIHC cohort, analyzed both in (**B**) unpaired samples and (**C**) matched tumor/non-tumor pairs. (**D**–**G**). Validation of *SLC25A39* upregulation in HCC using four independent GEO datasets: (**D**) GSE25097, (**E**) GSE63898, (**F**) GSE36376, and (**G**) GSE76427. Each panel shows significantly higher SLC25A39 levels in HCC tissues compared to non-tumor controls. (**H**). Representative immunohistochemistry images confirming elevated SLC25A39 protein expression in HCC tissue sections (**right**) compared to matched paracancerous non-tumor liver tissues (**left**) from six patients. Scale bar is provided (100 μm). *** *p* < 0.001.

Pan-cancer analysis of the XENA TCGA GTEx-ALL database revealed that *SLC25A39* mRNA expression is frequently upregulated across a wide range of tumor types (Figure 1A). Within the TCGA-LIHC cohort, *SLC25A39* was significantly overexpressed in HCC tissues compared to normal liver samples (Figure 1B). This upregulation was further confirmed in paired tumor and adjacent non-tumor tissues (Figure 1C). Consistent with this, analysis of four independent Gene Expression Omnibus (GEO) datasets (GSE25097, GSE63898, GSE36376, and GSE76427) consistently demonstrated higher *SLC25A39* mRNA levels in HCC (Figure 1D–G). Finally, immunohistochemical staining of patient-derived tissue sections validated the increased SLC25A39 protein expression in HCC compared to matched non-tumor liver tissues (Figure 1H). Collectively, these multi-omics and experimental data establish *SLC25A39* as a gene commonly upregulated in HCC, which is consistent with recent findings [10,11,12,13].

### 2.2. The Diagnostic Value and Prognostic Value of SLC25A39 in HCC

**Figure 2:**
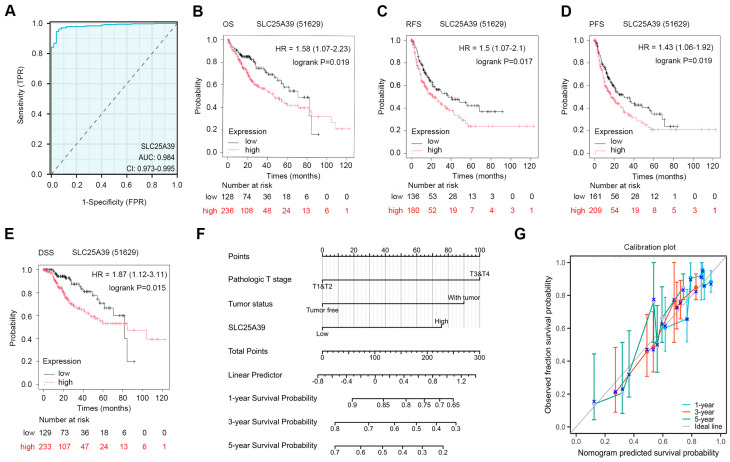
**The diagnostic value and prognostic value of *SLC25A39* in HCC.** (**A**). ROC analysis to evaluate the diagnostic value of *SLC25A39* in HCC. (**B**–**E**). Kaplan–Meier analysis demonstrates the prognostic value of *SLC25A39* for predicting (**B**) OS, (**C**) RFS, (**D**) PFS, and (**E**) DSS in HCC patients. (**F**). Nomogram for predicting 1-, 3-, and 5-year OS. (**G**). The calibration plots were used to evaluate the predictive accuracy of the nomogram. The blue cross on each line represents the result after stratified Kaplan-Meier correction for each point.

Analysis of the TCGA cohort revealed that high *SLC25A39* expression was significantly associated with advanced disease features, including higher pathologic T stage (*p* = 0.002), advanced overall pathologic stage (pathologic stage, tumor status, residual tumor) and elevated AFP level (Table 1). *SLC25A39* expression demonstrated high diagnostic accuracy for distinguishing HCC from normal tissue, with an area under the Receiver Operating Characteristic (ROC) curve (AUC) of 0.984 (Figure 2A). Kaplan–Meier survival analysis indicated that elevated *SLC25A39* expression was significantly correlated with poorer patient outcomes, manifested as shorter overall survival (OS; the time from diagnosis to death from any cause), relapse-free survival (RFS; the time without recurrence of the disease), progression-free survival (PFS; the time without tumor growth or spread), and disease-specific survival (DSS; the time to death specifically from HCC) (Figure 2B–E). A prognostic nomogram (a graphical predictive model) integrating *SLC25A39* expression with key clinical variables was developed to predict 1-, 3-, and 5-year survival probabilities, with its predictive accuracy supported by calibration plots (which compare predicted versus observed outcomes) (Figure 2F,G). Furthermore, univariate and multivariate Cox regression analyses (statistical models that evaluate the influence of variables on survival time) confirmed high *SLC25A39* expression as an independent risk factor for poorer overall survival in HCC patients (Table 2).

### 2.3. The Association Between SLC25A39 DNA Methylation and Prognosis in HCC

**Figure 3:**
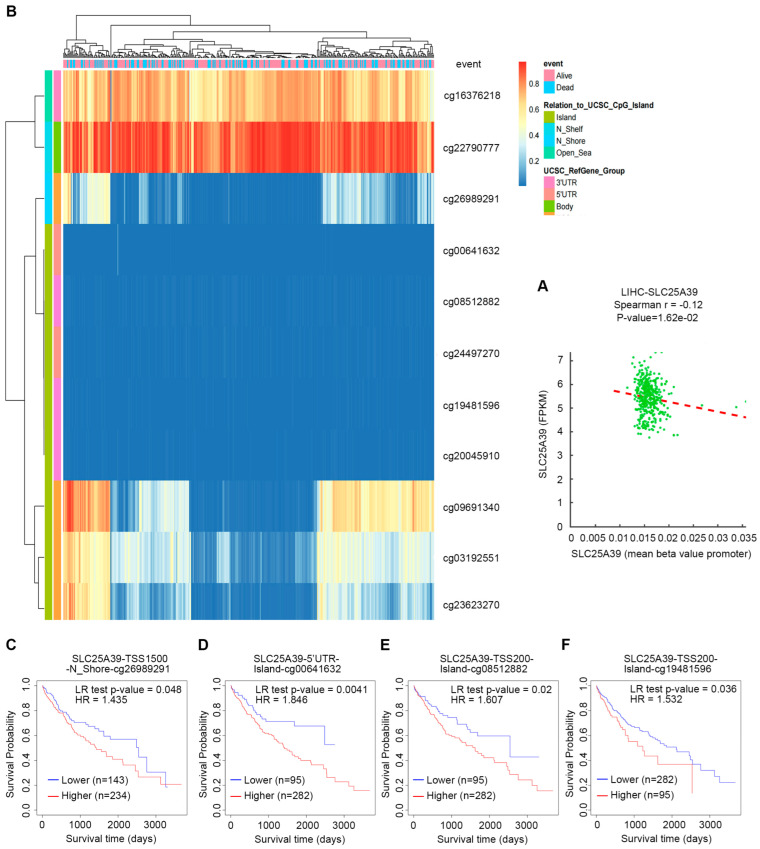
**The association between *SLC25A39* DNA methylation and prognosis in HCC.** (**A**) Correlation analysis between CpG methylation and SLC25A39 mRNA in DNA Methylation Interactive Visualization Database (DNMIVD). (**B**) Eleven CpG methylated sites in SLC25A39 were identified from the MethSurv database. (**C**) Association between SLC25A39 mRNA expression and its DNA methylation levels from the MethSurv database. (**C**–**F**). Association between SLC25A39 methylation level and overall survival of HCC. Kaplan–Meier survival curve of SLC25A39 CpG methylated sites in (**C**) cg26989291, (**D**) cg00641632, (**E**) cg08512882, (**F**) cg19481596.

Recent studies have underscored the significance of DNA methylation biomarkers for diagnosing and predicting outcomes in HCC, which also elucidate the molecular mechanisms that drive HCC progression [18]. We analyzed the DNA methylation profile of *SLC25A39* in HCC. A significant negative correlation was observed between *SLC25A39* promoter CpG methylation and its mRNA expression levels (r = −0.12, *p* = 0.0162; Figure 3A). Analysis of the MethSurv database identified 11 methylated CpG sites within the *SLC25A39* promoter region (Figure 3B). Among these, hypermethylation at four sites (cg26989291, cg00641632, cg08512882, cg19481596) was significantly associated with poorer overall survival in HCC patients (Figure 3C–F), while the remaining seven sites showed no significant prognostic value (Figure S1).

### 2.4. Enrichment Analysis of the Functions Associated with SLC25A39 Expression in HCC

**Figure 4:**
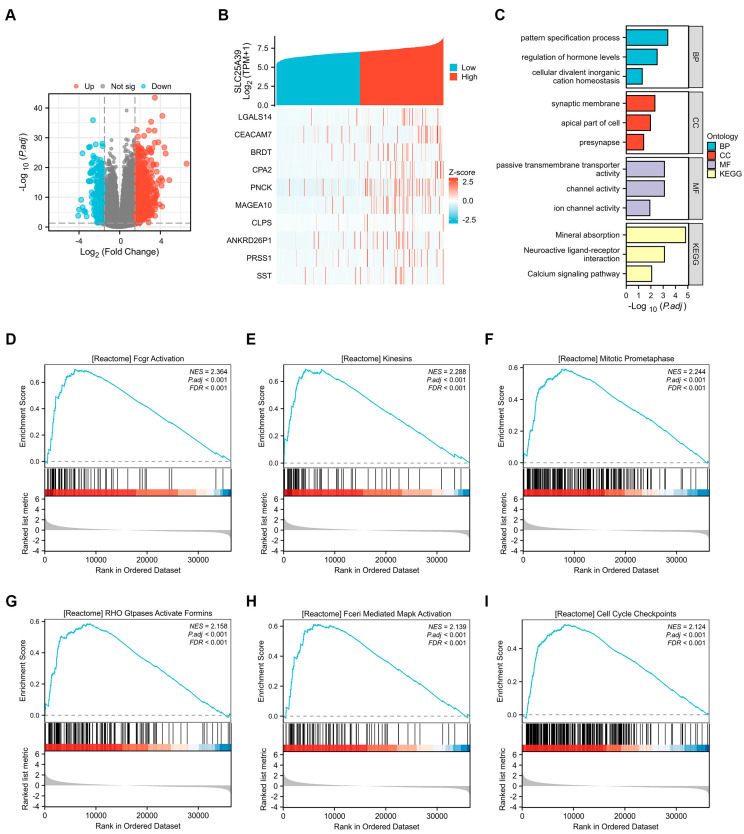
**Enrichment analysis of the functions associated with *SLC25A39* expression in HCC.** (**A**). Volcano plot displaying genes differentially expressed in *SLC25A39*-high versus *SLC25A39*- low HCC tumors from the TCGA database. (**B**). Visualization of the top 10 upregulated DEGs linked to *SLC25A39* expression by heatmap. (**C**). GO (including BP, CC, MF) and KEGG analyses of DEGs of *SLC25A39*. (**D**–**I**). Enrichment results of the GSEA gene set in thet *SLC25A39* high-expression group (top 6).

To investigate the potential biological functions of *SLC25A39* in HCC, we performed differential expression analysis on TCGA data. This identified 1120 differentially expressed genes between *SLC25A39*-high and SLC25A39-low tumors (911 up-, 209 downregulated; Figure 4A). The top 10 upregulated differentially expressed genes (DEGs) associated with *SLC25A39* in HCC are visualized by a heatmap, including *LGALS14*, *CEACAM7*, *BRDT*, *CPA2*, *PNCK*, *MAGEA10*, *CLPS*, *ANKRD26P1*, *PRSS1* and *SST* (Figure 4B). Functional enrichment analysis of these genes revealed significant associations with key biological themes. Gene Ontology (GO) analysis identified pattern specification processes, regulation of hormone levels, and cellular divalent inorganic cation homeostasis as the primary biological processes (BP). For cellular components (CCs), significant terms included the synaptic membrane, the apical part of the cell, and the presynapse. Key molecular functions (MF) were predominantly passive transmembrane transporter activity, channel activity, and ion channel activity. Concurrently, Kyoto Encyclopedia of Genes and Genomes (KEGG) pathway analysis further showed significant enrichment in mineral absorption, neuroactive ligand–receptor interaction, and the calcium signaling pathway (Figure 4C). Gene set enrichment analysis (GSEA) demonstrated that high *SLC25A39* expression was positively correlated with multiple tumor-promoting pathways, including fcgr activation, kinesins, mitotic prometaphase, Rho Gtpases activating formins, fceri mediated mapk activation, and cell cycle checkpoints regulation (Figure 4D–I).

### 2.5. Protein–Protein Interaction (PPI) Network for SLC25A39-Associated Genes

**Figure 5:**
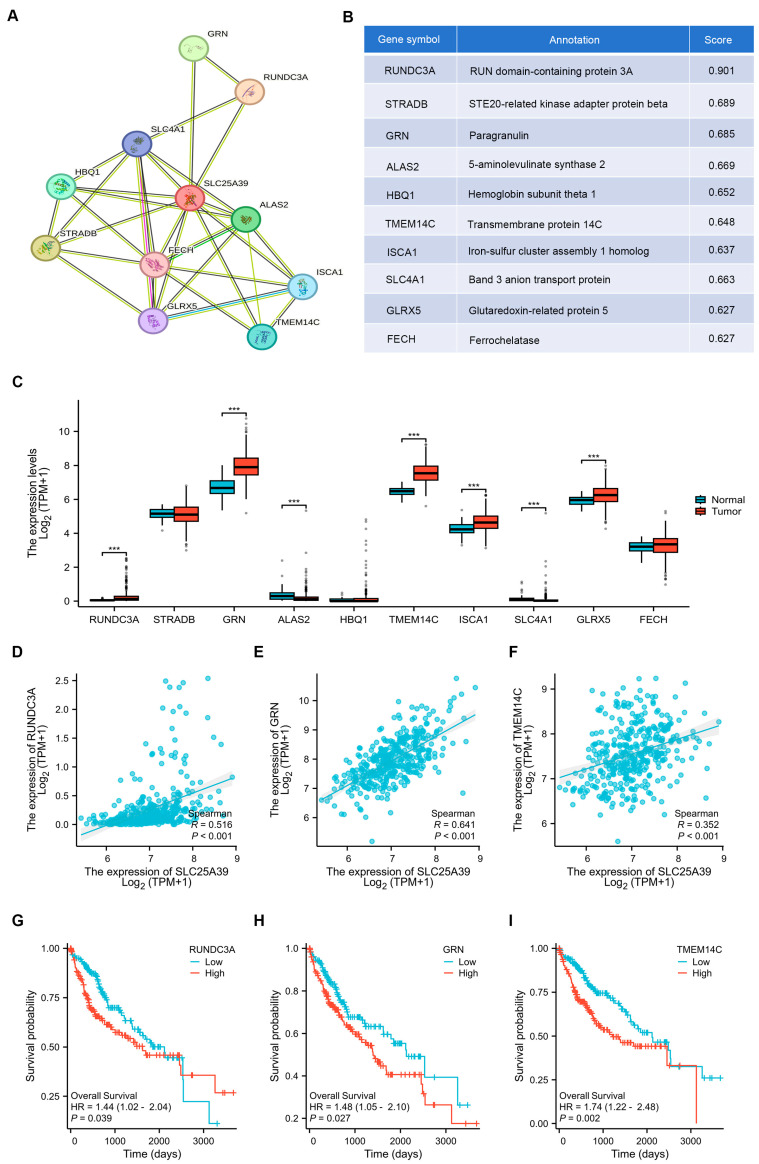
**Protein–protein interaction (PPI) network for *SLC25A39*-associated genes.** (**A**) PPI network for *SLC25A39*-associated genes from the STRING database. (**B**) Functional annotation and correlation coefficients of the top 10 *SLC25A39*-associated genes. (**C**) Relative mRNA expression of the top 10 *SLC25A39*-associated genes in HCC from TCGA database. (**D**–**F**). Correlation between *SLC25A39* and (**D**) *RUNDC3A*, (**E**) *GRN*, and (**F**) *TMEM14C* in HCC from TCGA database. (**G**–**I**). Overall survival of (**G**) *RUNDC3A*, (**H**) *GRN*, and (**I**) *TMEM14C* in HCC from TCGA database. *** *p* < 0.001.

Analysis of the Search Tool for the Retrieval of Interacting Genes (STRING) database identified the top 10 genes significantly associated with *SLC25A39*, constructing a protein–protein interaction (PPI) network (Figure 5A,B). Examination of these genes’ expression in the TCGA-LIHC cohort revealed differential expression patterns in HCC compared to adjacent non-tumor tissues, among which five genes (*RUNDC3A*, *GRN*, *TMEM14C*, *ISCA1*, *and GLRX5*) were upregulated in HCC, two genes (ALAS2 and SLC4A1) were downregulated, and three genes (*STRADB*, *HBQ1*, and *FECH*) were not significantly changed (Figure 5C). Correlation analysis indicated that among these interactors, SLC25A39 expression showed significant positive correlations with *RUNDC3A*, *GRN* and *TMEM14C* (Figure 5D–F), and a significant negative correlation with SLC4A1 (Figure S2C), but had no significant correlation with *ISCA1*, *GLRX5* or *ALAS2* (Figure S2A,B,D). Notably, elevated expression of *RUNDC3A*, *GRN*, and *TMEM14C* was each independently associated with poorer overall survival in HCC patients (Figure 5G–I), while the OS of HCC patients had no significant correlation with *ISCA1*, *GLRX5*, *SLC4A1*, and *ALAS2* (Figure S2E–H). This integrative analysis highlights *RUNDC3A*, *GRN*, and *TMEM14C* as key *SLC25A39*-associated genes that are co-upregulated in HCC and linked to adverse prognosis.

### 2.6. Correlation Between Immune Infiltration and SLC25A39 Expression in HCC

**Figure 6:**
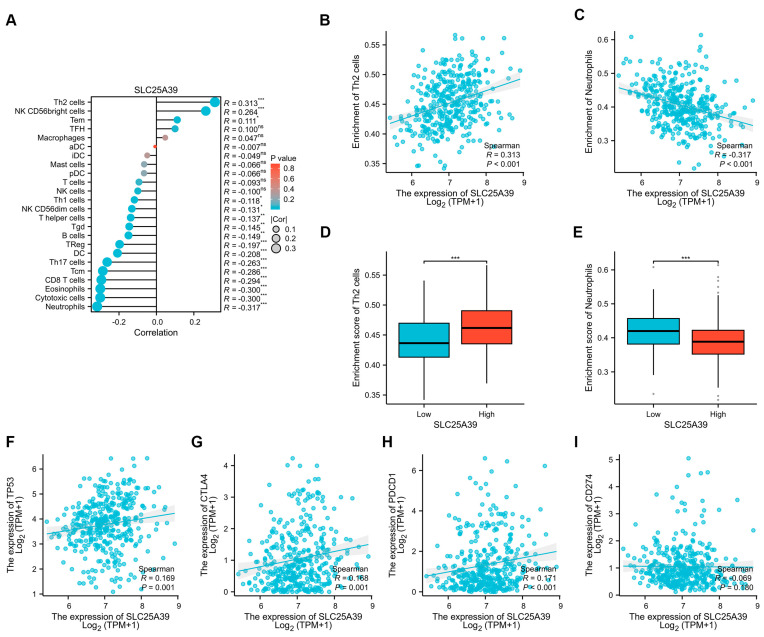
**Correlation between immune infiltration and *SLC25A39* expression in HCC.** (**A**) Correlation between *SLC25A39* expression and the estimated infiltration levels of 24 immune cell types. Immune cell enrichment scores were calculated using the single-sample gene set enrichment analysis (ssGSEA) algorithm based on established gene signatures. Spearman’s correlation coefficient (rho) and the corresponding *p*-value are labeled for each cell type. (**B**,**C**). Scatter plots showing the correlation between *SLC25A39* expression and the infiltration levels of (**B**) Th2 cells and (**C**) neutrophils, as representative examples. (**D**,**E**). Comparison of (**D**) Th2 cell and (**E**) neutrophil infiltration scores between HCC patients with high vs. low *SLC25A39* expression (defined by median cutoff). Statistical significance was assessed by the two-sided Wilcoxon rank-sum test. (**F**–**I**). Scatter plots depicting the correlations of *SLC25A39* expression with (**F**) *TP53*, (**G**) *CTLA-4*, (**H**) *PD-1*, and (**I**) *PD-L1* mRNA expression. Spearman’s rho and *p*-value are indicated in each panel. ns, not significant, * *p* < 0.05, ** *p* < 0.01, *** *p* < 0.001.

To computationally assess the potential role of *SLC25A39* in the tumor immune microenvironment (TIME) of HCC, we employed the single-sample gene set enrichment analysis (ssGSEA) algorithm to estimate the enrichment scores of 24 immune cell populations based on bulk tumor transcriptomic data. *SLC25A39* expression exhibited distinct correlations with specific immune subsets. It showed significant positive correlations with the infiltration of Th2 cell (R = 0.313, *p* < 0.001), NK CD56bright cells (R = 0.264, *p* < 0.001) and Tem (R = 0.111, *p* < 0.001). Conversely, *SLC25A39* expression was negatively correlated with the infiltration of multiple immune cell types, most strongly with neutrophils (R = −0.317, *p* < 0.001), cytotoxic cells (R = −0.300, *p* < 0.001), eosinophils (R = −0.300, *p* < 0.001), CD8 T cells (R = −0.294, *p* < 0.001), Tcm (R = −0.286, *p* < 0.001), Th17 cells (R = −0.263, *p* < 0.001), DC (R = −0.208, *p* < 0.001), Treg (R = −0.197, *p* < 0.001), B cells (R = −0.149, *p* < 0.01), Tgd (R = −0.145, *p* < 0.01), T helper cells (R = −0.137, *p* < 0.01), NK CD56dim cells (R = −0.131, *p* < 0.05), and Th1 cells (R = −0.118, *p* < 0.05) (Figure 6A–C). Accordingly, HCC patients with high *SLC25A39* expression were associated with increased Th2 cells infiltration and reduced neutrophil infiltration (Figure 6D–E). Further analysis of T-cell subsets revealed a significant negative correlation between *SLC25A39* expression and the Th17 lineage marker *RORC* (R = −0.211, *p* < 0.001) but no significant correlation with the Treg marker *FOXP3* (R = −0.069, *p* = 0.183) (Figure S3A,B), suggesting a more specific association with Th17 cell regulation in the HCC microenvironment. Given the established links between *TP53* mutation, immune checkpoint expression, and HCC progression [14,19], we evaluated their relationship with *SLC25A39*. *SLC25A39* expression showed significant positive correlations with *TP53* (R = 0.169, *p* < 0.001) and the immune checkpoint genes *CTLA-4* (R = 0.168, *p* < 0.001) and *PD-1* (*PDCD-1*; R = 0.171, *p* < 0.001) but not with *PD-L1* (*CD274*; R = −0.069, *p* = 0.180) (Figure 6F–I). These results suggest *SLC25A39* within an immunosuppressive HCC microenvironment characterized by altered immune cell composition and elevated checkpoint molecule expression.

### 2.7. Correlation Between Drug Sensitivity and SLC25A39 Expression

**Figure 7:**
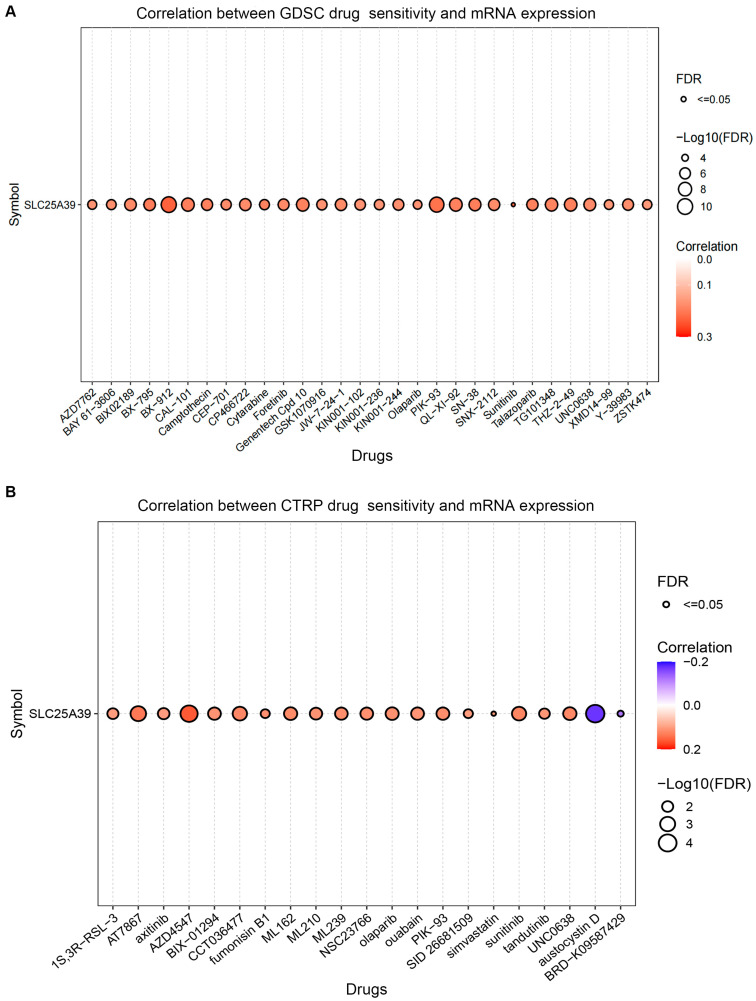
**Pan-cancer analysis of the correlation between *SLC25A39* and drug sensitivity.** (**A**,**B**) Pan-cancer analysis of the correlation between *SLC25A39* expression and drug sensitivity. Data were derived from the GSCALite database integrating (**A**) GDSC and (**B**) CTRP datasets. The top 30 significant correlations (based on FDR) are shown. The color of the bubble represents Spearman’s correlation coefficient; all the presented drugs denote correlations with an FDR ≤ 0.05. Complete data for all correlated drugs, including correlation coefficients and FDR values, are provided in Tables S1 and S2. Correlation between *SLC25A39* expressions and drug sensitivity from the GDSC database. (**B**) Relationship between *SLC25A39* expressions and drug sensitivity from the CTRP database.

To explore the translational potential of *SLC25A39*, we analyzed its association with drug sensitivity using the GSCALite platform, which integrates data from the Genomics of Drug Sensitivity in Cancer (GDSC) and Cancer Therapeutics Response Portal (CTRP) databases. Pan-cancer analysis revealed that elevated *SLC25A39* expression was significantly correlated with resistance to multiple pharmacological agents. In the GDSC dataset, the most prominent correlations were observed with BX-912 (Cor = 0.232239335, FDR = 7.34516 × 10^−12^), Sunitinib (Cor = 0.222525592, FDR = 2.43132 × 10^−4^), and PIK-93 (Cor = 0.212030486, FDR = 4.65257 × 10^−10^) (Figure 7A, Table S1). In the CTRP dataset, the top three medications positively correlated with *SLC25A39* expression were AZD4547 (Cor = 0.159682029, FDR = 1.80339 × 10^−4^), AT7867 (Cor = 0.134738459, FDR = 6.80948 × 10^−4^), and Sunitinib (Cor = 0.123547684, FDR = 1.755769 × 10^−3^). Conversely, *SLC25A39* expression was significantly correlated with sensitivity to austocystin D (Cor = −0.170942458, FDR = 5.9835 × 10^−5^) and BRD-K09587429 (Cor = −0.120823593, FDR =0.040724936) (Figure 7B, Table S2). Conversely, *SLC25A39* expression was correlated with increased sensitivity to austocystin D and BRD-K09587429 in the CTRP dataset. This analysis extends the known association of *SLC25A39* with sorafenib resistance to a broader spectrum of therapeutics.

### 2.8. SLC25A39 Facilitates HCC Cell Proliferation, Migration and Invasion

**Figure 8:**
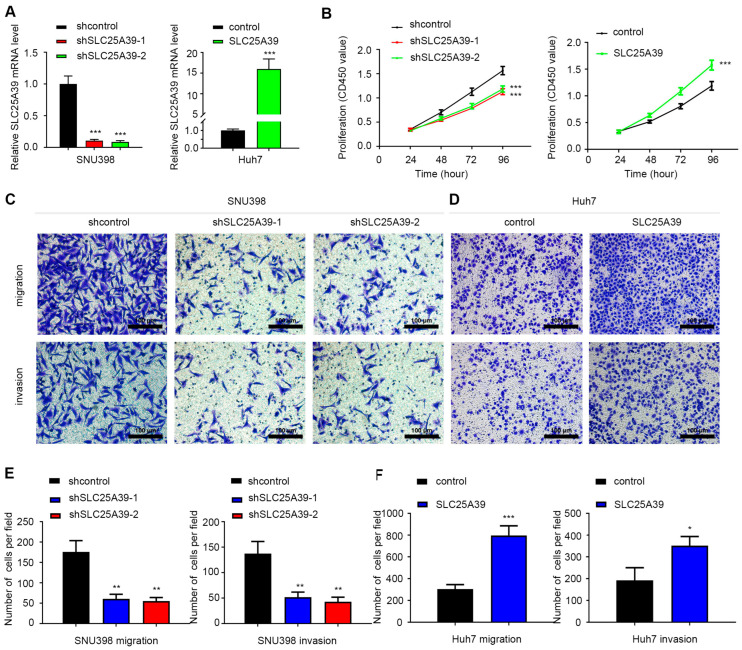
***SLC25A39* facilitates HCC cell proliferation, migration and invasion.** (**A**). Transfection efficiencies of *SLC25A39*-knockdown plasmid in SNU398- and *SLC25A39*-overexpressing plasmid in Huh7 were measured by qRT-PCR (n = 3). (**B**). The CCK-8 assays were conducted to assess cell proliferation (n = 6). (**C**,**D**). The Transwell assays were performed to measure cell migration and invasion (n = 3). (**E**,**F**). Quantification of the Transwell migration and invasion assay from panels (**C**). (**F**) Quantification of the Transwell migration and invasion assay from panels (**D**). The bar graph shows the mean number of migrated and invaded cells per field. Data are presented as mean ± SD. Statistical significance was determined by Student’s *t*-test. * *p* < 0.05, ** *p* < 0.01, *** *p* < 0.001, as compared to their respective control groups.

To experimentally validate the oncogenic function of *SLC25A39*, we performed loss- and gain-of-function studies in HCC cell lines. Based on expression profiles among 24 kinds of HCC cell lines from the Cancer Cell Line Encyclopedia (CCLE, Table 3), we selected SNU398 (relatively high endogenous *SLC25A39*) and Huh7 (relatively low endogenous *SLC25A39*) for functional assays. SNU398 cells were transfected with *SLC25A39*-knockdown plasmid, while Huh7 cells were transfected with *SLC25A39*-overexpressing plasmid constructs. Efficient knockdown of *SLC25A39* in SNU398 cells and its overexpression in Huh7 cells were confirmed (Figure 8A). CCK-8 assays demonstrated that *SLC25A39* knockdown significantly inhibited the proliferation of SNU398 cells, while its overexpression enhanced the proliferation of Huh7 cells (Figure 8B). Furthermore, Transwell assays revealed that *SLC25A39* depletion attenuated, whereas its overexpression promoted, the migratory and invasive capacities of SNU398 and Huh7 cells, respectively (Figure 8C–F). These in vitro findings functionally confirm the pro-tumorigenic role of SLC25A39 in HCC, reinforcing the established pro-tumorigenic role of *SLC25A39* in HCC [10,11,12,13].

## 3. Discussion

Our study builds upon and significantly extends the emerging understanding of *SLC25A39* in HCC pathogenesis. Consistent with prior reports linking *SLC25A39* to sorafenib resistance, mitochondrial ROS signaling, ferroptosis evasion, and necroptosis suppression [10,11,12,13], we confirmed its upregulation, association with poor prognosis, and pro-tumorigenic functions in vitro. Moving beyond these cell-intrinsic roles, our integrated analysis reveals novel dimensions of *SLC25A39* involving epigenetic regulation, immune microenvironment modulation, and extended therapeutic associations. Specifically, we provide several key novel insights into the biology of *SLC25A39* in HCC.

First, we provide the first evidence linking *SLC25A39* promoter DNA methylation to patient survival in HCC. Hypermethylation at four specific CpG sites (including cg26989291, cg00641632, cg08512882, and cg19481596) correlated with poorer outcomes, introducing an epigenetic layer to its regulation in line with the recognized importance of DNA methylation in HCC [18].

Second, and most significantly, our study pioneers the exploration of the relationship between *SLC25A39* and the TIME in HCC. While *SLC25A39* has been linked to immunotherapy resistance in colorectal cancer [17], its role in the HCC immune landscape was unknown. Although these bioinformatic findings do not establish a direct causal role of *SLC25A39* in regulating immune cell recruitment or function, our analyses suggest that high *SLC25A39* expression correlates with a distinct immunosuppressive contexture, reduced cytotoxic infiltration, and elevated checkpoint expression. Specifically, *SLC25A39* correlates with a Th2-skewed cytokine profile (favoring IL-4, IL-10) over a Th1 response, alongside diminished infiltration of key antitumor effector cells, including cytotoxic CD8^+^ T cells and neutrophils. As widely acknowledged, CD8^+^ T lymphocytes are central to antitumor immunity by directly targeting and eliminating cancer cells. In HCC, however, their infiltration and function are often suppressed, leading to an exhausted phenotype marked by impaired cytotoxicity and sustained expression of inhibitory receptors like PD-1 and CTLA-4. This dysfunction facilitates tumor immune evasion and progression. Therefore, understanding the regulators of CD8^+^ T cell activity in the HCC microenvironment is crucial for developing effective immunotherapies [14]. Neutrophils can exhibit tumoricidal activity in HCC via ROS secretion. Notably, and with direct translational implication, *SLC25A39* was positively correlated with the expression of critical immune checkpoint molecules *CTLA-4* and *PD-1*. The concurrent negative correlation with both Th17 and Treg infiltration presents an intriguing paradox, as Th17 cells (often pro-inflammatory) and Tregs (immunosuppressive) typically exert opposing functions in tumor immunity [14]. As *SLC25A39* was correlated with *RORC* rather than *FOXP3*, our study indicates *SLC25A39*’s association with Th17 cells might be more direct or pronounced than Treg. However, this complex relationship merits further study.

Third, functional enrichment analyses connect *SLC25A39* to biological processes like cation homeostasis and pathways such as mineral absorption, neuroactive ligand–receptor interaction and the calcium signaling pathway, which are implicated in HCC progression [20,21,22,23,24]. Briefly, the mineral absorption pathway is significantly correlated with HCC onset and progression [20], and metal ion metabolism substantially influences HCC development and therapy [21]. The neuroactive ligand–receptor interaction pathway comprises plasma-membrane-associated receptors and ligands involved in intra- and extracellular signaling, which were relevant to HCC progression [22]. Neuroactive ligand–receptor interactions have been observed across early, intermediate, and advanced HCC stages [23]. Dysregulation of calcium signaling, a critical pathway in cellular metabolism, has been linked to HCC development and progression [24]. GSEA revealed that DEGs of high *SLC25A39* expression were enriched in fcgr activation, kinesins, mitotic prometaphase, Rho Gtpases-activated formins, fceri-mediated mapk activation, and cell cycle checkpoints. As reported, kinesin superfamily proteins [25], mitotic prometaphase [26], Rho GTPase family [27], mapk activation [28], and cell cycle checkpoints [29] are involved in HCC pathogenesis. Furthermore, PPI network analysis highlighted *RUNDC3A*, *GRN*, and *TMEM14C* as key correlating genes; notably, *GRN* (also known as granulin-epithelin precursor, *GEP*) acts as an oncogene in HCC [30], and all three are linked to poor survival, suggesting a coordinated network. Thus, these results offer mechanistic avenues for future investigation that complement the pathways (e.g., Hedgehog, ferroptosis) identified by other studies [10,11,12,13].

Finally, drug sensitivity analysis extends *SLC25A39*’s role beyond sorafenib resistance [10,12], linking its expression to response to a broader spectrum of pharmacological agents (e.g., BX-912, PIK-93, Sunitinib). Intriguingly, several identified drugs have reported immunomodulatory effects [31,32,33]. For instance, the PDK inhibitor BX-912 can enhance the efficacy of anti-PD-L1 therapy in breast cancer [31]. The PI4K inhibitor PIK-93 reduces PD-L1 expression and synergizes with anti-PD-L1 antibodies and might be related to HCC treatment [32]. The FGFR inhibitor AZD4547 may alleviate immunosuppression in HCC [33]. Collectively, these results reinforce the potential intersection of *SLC25A39* activity with immune regulation.

While our study provides novel insights into the epigenetic regulation and immunomodulatory potential of *SLC25A39* in HCC, several limitations should be acknowledged. First, the expression and prognostic findings of *SLC25A39* need verification in large-scale, multicenter independent cohorts. Second, the mechanistic links suggested by our bioinformatics analyses remain correlative. Specifically, the associations between *SLC25A39* expression and the immunosuppressive tumor microenvironment (e.g., immune cell infiltration, checkpoint molecule expression) are derived from computational deconvolution of bulk transcriptomic data. Similarly, the link between *SLC25A39* promoter methylation and prognosis is observational. These findings do not establish causality. Future studies employing *SLC25A39*-modulated in vitro co-culture systems, detailed flow cytometry analysis of tumor-infiltrating lymphocytes, and in vivo immune-competent models are essential to validate these associations and elucidate the direct mechanistic role of *SLC25A39* in shaping the TIME and its epigenetic regulation. Third, the drug sensitivity correlations identified through database mining are predictive and necessitate functional validation in HCC models to assess their therapeutic relevance. Addressing these limitations in future studies will be vital to translate our findings into clinically actionable strategies.

## 4. Materials and Methods

### 4.1. Analyses of SLC25A39 Expression in Public Databases

Firstly, we examined the expression profile of *SLC25A39* across various cancer types using the XENA TCGA GTEx-ALL database (https://xenabrowser.net/datapages/, accessed on 19 January 2025). Subsequently, its expression was evaluated in the HCC cohort from The Cancer Genome Atlas (TCGA, https://portal.gdc.cancer.gov, accessed on 19 January 2025) via ggplot2 (v3.4.4), which included 50 normal and 374 tumor tissues along with associated clinicopathological data [34]. We further assessed *SLC25A39* mRNA levels in four Gene Expression Omnibus (GEO) datasets (GSE25097, GSE63898, GSE36376, and GSE76427) (https://www.ncbi.nlm.nih.gov/geo/, accessed on 19 January 2025) [35]. For GEO datasets from different platforms or batches, the ComBat function from the “sva” R package (version 3.42.0) was used to correct for batch effects and remove technical variations. Batch effects were visualized via Principal Component Analysis (PCA) before correction, and the effectiveness of correction was confirmed post-correction [34]. As the clinical data were obtained from publicly available sources, informed consent was presumed to have been previously acquired.

### 4.2. Immunohistochemistry (IHC) Staining

Liver tissue samples, including HCC and adjacent non-tumor tissues (collected at least 2 cm from the tumor margin), were obtained from patients who underwent surgical resection at Tongji Hospital, Tongji Medical College, Huazhong University of Science and Technology (Wuhan, China) between June 2021 and December 2022. The tissues were preserved in 4% paraformaldehyde (Servicebio, Wuhan, China), followed by paraffin embedding and sectioning into 5 μm slices. Immunohistochemistry (IHC) staining was performed as previously outlined [34], using a primary antibody against SLC25A39 (14963-1-AP, Proteintech, Wuhan, China) at a dilution of 1:100. The study was approved by the Institutional Ethics Committee of Tongji Hospital (Approval No. TJ-C20210601, Date: 1 June 2021), and all procedures adhered to the Declaration of Helsinki. Written informed consent was acquired from all participating patients.

### 4.3. The Clinical Significance of SLC25A39 in HCC

To evaluate the clinical relevance of *SLC25A39* in HCC, tumor and adjacent normal tissues from 374 patients and 50 controls in the TCGA HCC cohort were stratified into high- and low-expression groups according to the median *SLC25A39* mRNA expression. Associations between *SLC25A39* expression and clinicopathological features—including gender, race, T/N/M stage, histologic grade, pathologic stage, and tumor status—were assessed using the chi-square test [34]. The diagnostic potential of *SLC25A39* was evaluated via Receiver Operating Characteristic (ROC) curve analysis [34]. For prognostic assessment, Cox regression and Kaplan–Meier survival analyses (https://kmplot.com/analysis/, accessed on 24 January 2025) were employed [34]. Additionally, a nomogram was developed using the RMS R package (v6.3-0) [36] to predict overall survival (OS) in HCC patients.

### 4.4. DNA Methylation Analysis

Correlation between CpG methylation and *SLC25A39* mRNA expression in HCC was analyzed in the DNA Methylation Interactive Visualization Database (DNMIVD, http://www.unimd.org/dnmivd, accessed on 26 January 2025) [37]. The prognostic significance of *SLC25A39* DNA methylation sites in HCC was evaluated using MethSurv (https://biit.cs.ut.ee/methsurv/, accessed on 26 January 2025), a web-based tool designed for DNA methylation-based survival analysis [38].

### 4.5. Analysis of Functional Enrichment

Differentially expressed genes (DEGs) between patients exhibiting high and low levels of *SLC25A39* expression were identified using the DESeq2 package [39]. Genes meeting the criteria of an adjusted *p*-value < 0.05 and an absolute fold change (FC) > 1.5 were selected for further investigation. Functional annotation of *SLC25A39*-associated DEGs was carried out through Gene Ontology (GO) enrichment analysis, covering the categories of biological processes (BPs), cellular components (CCs), and molecular functions (MFs). Pathway analysis was subsequently performed using the Kyoto Encyclopedia of Genes and Genomes (KEGG) to identify signaling pathways linked to these DEGs. For both GO and KEGG enrichment analyses, terms with an adjusted *p*-value (False Discovery Rate, FDR) of less than 0.05 were considered statistically significant [40]. Additionally, gene set enrichment analysis (GSEA) was employed to explore biological pathways in patients with high *SLC25A39* expression [41]. Significance thresholds were set at an adjusted *p*-value < 0.05 and an FDR < 0.25 after 1000 permutation tests. All statistical computations and visualizations were conducted using the ClusterProfiler and ggplot2 packages in R [v3.3.6] [41]. A protein–protein interaction (PPI) network was constructed via the Search Tool for the Retrieval of Interacting Genes (STRING) database, and the top 10 interaction networks were visualized using the igraph R package (v1.4.1) [42]. Furthermore, the expression and prognostic relevance of genes correlated with *SLC25A39* in HCC were assessed based on TCGA data, along with an evaluation of their correlation with SLC25A39.

### 4.6. Immune Infiltration Analysis

The relative infiltration levels (enrichment scores) of 24 immune cell types in the HCC tumor microenvironment were estimated using the single-sample gene set enrichment analysis (ssGSEA) algorithm, as implemented in the GSVA R package (ggplot2 [3.4.4], R version 4.2.1) [43]. This method quantifies the activity of predefined gene signatures, specific to each immune cell type, within each bulk tumor transcriptomic sample. The gene signatures for immune cell characterization were based on a widely used and established resource [44]. To assess the relationship between *SLC25A39* expression and the immune landscape, Spearman’s rank correlation analysis was performed between *SLC25A39* expression and the ssGSEA-derived immune cell enrichment scores, as well as the mRNA expression levels of key immune checkpoint molecules (*CTLA-4*, *PDCD1/PD1*, *CD274/PDL1*) and *TP53*. The strength and significance of these monotonic relationships are reported using Spearman’s rho (ρ) coefficient and the corresponding *p*-value. Furthermore, differences in immune cell infiltration scores between patient groups stratified by high or low *SLC25A39* expression (based on the median cutoff) were compared using the Wilcoxon rank-sum test.

### 4.7. Analysis of Drug Sensitivity

The relationship between *SLC25A39* expression and drug sensitivity was investigated through GSCALite (https://guolab.wchscu.cn/GSCA, accessed on 1 March 2026) utilizing drug response data from Genomics of Drug Sensitivity in Cancer (GDSC) and Cancer Therapeutics Response Portal (CTRP) [45]. The data was examined by calculating Spearman’s correlation coefficients. A positive correlation signified that elevated gene expression was linked to drug resistance, whereas lower expression conferred sensitivity to the drug.

### 4.8. Cell Culture

The HCC cell lines SNU398 and Huh7 were utilized in this study. SNU398 was acquired from the American Type Culture Collection (Manassas, VA, USA), and Huh7 was sourced from the Japanese Cancer Research Bank (Tokyo, Japan); both lines were maintained in our laboratory. SNU398 cells were grown in RPMI-1640 medium (GIBCO, Waltham, MA, USA), and Huh7 cells were propagated in DMEM (GIBCO). Each culture medium was supplemented with 10% fetal bovine serum (GIBCO) and 1% penicillin/streptomycin (Servicebio). All cells were incubated at 37 °C in a humidified atmosphere containing 5% CO_2_ [34].

### 4.9. Transfection of Plasmid and Quantitative Reverse Transcription Polymerase Chain Reaction (qRT-PCR)

Knockdown (designated as *shSLC25A39*) and overexpression (designated as *SLC25A39*) plasmids for *SLC25A39*, along with their corresponding control plasmids (respectively designated as shcontrol and control), were acquired from Genomeditech Co., Ltd. (Shanghai, China). The targeting sequences for the shRNAs were shRNA-1, 5′-TCTACCCTTTGACGTGGTAAA-3′; shRNA-2, 5′-CTGGAGCTTATGCGGACAAAG -3′, as previously described [46]. SNU398 cells were transfected with the knockdown plasmid (shRNA), while Huh7 cells were transfected with the *SLC25A39*-overexpressing plasmid. At 48 h post-transfection, total mRNA was isolated from the cells using TRIzol reagent (Invitrogen, Carlsbad, CA, USA). As outlined in Section 4, first-strand cDNA synthesis was carried out with a PrimeScript reagent kit (Takara, Dalian, China). Subsequently, real-time PCR analysis of the target genes was performed to verify the transfection efficiency using SYBR Premix ExTaq (Takara) in strict accordance with the manufacturer’s protocols [46]. The primer sequences used for qRT-PCR are listed as follows: *SLC25A39*: Forward 5′-CCCTGGAGCTTATGCGGAC-3′, Reverse 5′-GCCTGAACCCATTGAGCCA-3′, *GAPDH*: Forward 5′-GCACCGTCAAGGCTGAGAAC-3′, Reverse 5′-TGGTGAAGACGC CAGTGGA-3′.

### 4.10. Cell Proliferation Analyses

The proliferative capacity of SNU398 and Huh7 cells was assessed using a Cell Counting Kit-8 (CCK-8) assay (Beyotime, Shanghai, China). Briefly, 1000 cells per well were plated in 96-well plates and cultured under standard conditions. Following the previous protocol [46], at designated time points (24, 48, 72, and 96 h), the cells were gently rinsed three times with PBS and subsequently incubated for 2 h in a mixture of 100 μL complete medium and 10 μL CCK-8 reagent (Promoter Biotechnology, Wuhan, China) at 37 °C. Optical density (OD) at 450 nm was finally quantified using a microplate reader (Biotek, Winooski, VT, USA). The experiment was independently repeated six times (n = 6 biological replicates). Data are presented as the mean ± standard deviation (SD). Statistical significance between groups at each time point was determined using the unpaired, two-tailed Student’s *t*-test. A *p*-value < 0.05 was considered statistically significant.

### 4.11. Cell Migration and Invasion Analyses

The Transwell assay was utilized to quantify cell migration and invasion, following a previously described method [46]. Briefly, Transwell chambers (8 μm pore size, Corning, NY, USA) were either pre-coated with Matrigel (BD Bioscience, Franklin Lakes, NJ, USA) for invasion assays or used uncoated for migration assays. A total of 5 × 10^4^ (migration) or 1 × 10^5^ (invasion) cells in 200 μL serum-free DMEM were seeded into the upper chamber, while the lower chamber was filled with 600 μL complete DMEM to serve as a chemoattractant. Following a 24 h incubation, the non-migratory cells were scraped off the upper membrane surface. The migrated/invaded cells on the lower surface were fixed with 4% paraformaldehyde (Servicebio) and stained with crystal violet (Beyotime, Shanghai, China). Microscopic observation (Olympus, Hachioji, Tokyo, Japan) and cell counting were conducted in a minimum of three predefined fields. Each experiment was performed in triplicate wells and independently repeated three times (n = 3 biological replicates). Data are presented as the mean number of cells per field ± SD. Differences between groups were analyzed using the unpaired, two-tailed Student’s *t*-test, with *p* < 0.05 considered statistically significant.

### 4.12. Statistical Analysis

All statistical analyses were performed using GraphPad Prism 10 (San Diego, CA, USA) and SPSS 19.0 (Chicago, IL, USA). The non-parametric Wilcoxon rank-sum test was used to compare *SLC25A39* expression between unpaired groups, and the paired-sample *t*-test was used for paired comparisons. Associations between dichotomized *SLC25A39* expression (high/low) and clinicopathological features were assessed by logistic regression, reporting odds ratios (ORs) with 95% confidence intervals (CIs). Correlations between continuous variables were evaluated using Spearman’s rank correlation, reporting the coefficient (R). A *p*-value of less than 0.05 was considered statistically significant. To control for multiple testing in transcriptomic analyses, the False Discovery Rate (FDR) was applied. For differential gene expression (DESeq2), FDR < 0.05 and |fold change| > 1.5 defined significance.

## 5. Conclusions

In conclusion, our study positions SLC25A39 not only as a driver of tumor cell-intrinsic phenotypes but also as a potential regulator of the immunosuppressive landscape in HCC. Targeting SLC25A39 could therefore represent a dual-pronged therapeutic strategy, simultaneously impairing tumor cell viability and dismantling immune evasion mechanisms, offering a compelling rationale for exploring SLC25A39 inhibition in combination strategies.

## Figures and Tables

**Table 1:** The association between *SLC25A39* expression and clinicopathological characteristics.

Characteristics	Low Expression of SLC25A39	High Expression of SLC25A39	*p* Value
n	187	187	
Gender, n (%)			0.740
Female	59 (15.8%)	62 (16.6%)	
Male	128 (34.2%)	125 (33.4%)	
Race, n (%)			0.202
Asian	83 (22.9%)	77 (21.3%)	
Black or African American	5 (1.4%)	12 (3.3%)	
White	95 (26.2%)	90 (24.9%)	
Age, n (%)			0.639
≤60	91 (24.4%)	86 (23.1%)	
>60	96 (25.7%)	100 (26.8%)	
Height, n (%)			0.761
<170	100 (29.3%)	101 (29.6%)	
≥170	72 (21.1%)	68 (19.9%)	
Weight, n (%)			0.275
≤70	88 (25.4%)	96 (27.7%)	
>70	87 (25.1%)	75 (21.7%)	
BMI, n (%)			0.859
≤25	89 (26.4%)	88 (26.1%)	
>25	82 (24.3%)	78 (23.1%)	
Pathologic T stage, n (%)			0.002
T1	109 (29.4%)	74 (19.9%)	
T2	40 (10.8%)	55 (14.8%)	
T3	33 (8.9%)	47 (12.7%)	
T4	3 (0.8%)	10 (2.7%)	
Pathologic N stage, n (%)			0.560
N0	133 (51.6%)	121 (46.9%)	
N1	1 (0.4%)	3 (1.2%)	
Pathologic M stage, n (%)			1.000
M0	139 (51.1%)	129 (47.4%)	
M1	2 (0.7%)	2 (0.7%)	
Pathologic stage, n (%)			0.005
Stage I	103 (29.4%)	70 (20%)	
Stage II	37 (10.6%)	50 (14.3%)	
Stage III	33 (9.4%)	52 (14.9%)	
Stage IV	3 (0.9%)	2 (0.6%)	
Tumor status, n (%)			0.043
Tumor free	113 (31.8%)	89 (25.1%)	
With tumor	69 (19.4%)	84 (23.7%)	
Residual tumor, n (%)			0.005
R0	172 (49.9%)	155 (44.9%)	
R1	3 (0.9%)	14 (4.1%)	
R2	1 (0.3%)	0 (0%)	
Histologic grade, n (%)			0.056
G1	32 (8.7%)	23 (6.2%)	
G2	97 (26.3%)	81 (22%)	
G3	50 (13.6%)	74 (20.1%)	
G4	6 (1.6%)	6 (1.6%)	
Child-Pugh grade, n (%)			0.496
A	115 (47.7%)	104 (43.2%)	
B	13 (5.4%)	8 (3.3%)	
C	1 (0.4%)	0 (0%)	
Fibrosis ishak score, n (%)			0.369
0	41 (19.1%)	34 (15.8%)	
1/2	13 (6%)	18 (8.4%)	
3/4	15 (7%)	13 (6%)	
5&6	49 (22.8%)	32 (14.9%)	
Adjacent hepatic tissue inflammation, n (%)			0.664
None	66 (27.8%)	52 (21.9%)	
Mild	56 (23.6%)	45 (19%)	
Severe	12 (5.1%)	6 (2.5%)	
Vascular invasion, n (%)			0.108
No	118 (37.1%)	90 (28.3%)	
Yes	52 (16.4%)	58 (18.2%)	
Albumin(g/dL), n (%)			0.729
<3.5	36 (12%)	33 (11%)	
≥3.5	126 (42%)	105 (35%)	
AFP(ng/mL), n (%)			0.022
≤400	124 (44.3%)	91 (32.5%)	
>400	27 (9.6%)	38 (13.6%)	

**Table 2:** Univariate and multivariate analyses of clinicopathological parameters in patients with HCC in TCGA–LIHC.

Characteristics	Total (N)	Univariate Analysis		Multivariate Analysis	
		Hazard Ratio (95% CI)	*p* Value	Hazard Ratio (95% CI)	*p* Value
Pathologic T stage	370				
T1&T2	277	Reference		Reference	
T3&T4	93	2.598 (1.826–3.697)	<0.001	1.788 (1.028–3.110)	0.040
Histologic grade	368				
G1&G2	233	Reference			
G3&G4	135	1.091 (0.761–1.564)	0.636		
Tumor status	354				
Tumor free	202	Reference		Reference	
With tumor	152	2.317 (1.590–3.376)	<0.001	1.893 (1.188–3.017)	0.007
Age	373				
≤60	177	Reference			
>60	196	1.205 (0.850–1.708)	0.295		
AFP(ng/mL)	279				
≤400	215	Reference			
>400	64	1.075 (0.658–1.759)	0.772		
Pathologic stage	349				
Stage I	173	Reference		Reference	
Stage II and Stage III and Stage IV	176	2.090 (1.429–3.055)	<0.001	1.537 (0.841–2.808)	0.163
Vascular invasion	317				
No	208	Reference			
Yes	109	1.344 (0.887–2.035)	0.163		
Gender	373				
Female	121	Reference			
Male	252	0.793 (0.557–1.130)	0.200		
Race	361				
Asian	159	Reference			
Black or African American and White	202	1.341 (0.926–1.942)	0.121		
Height	340				
<170	201	Reference			
≥170	139	1.232 (0.849–1.788)	0.272		
Weight	345				
≤70	184	Reference			
>70	161	0.941 (0.657–1.346)	0.738		
BMI	336				
≤25	177	Reference			
>25	159	0.798 (0.550–1.158)	0.235		
Pathologic N stage	258				
N0	254	Reference			
N1	4	2.029 (0.497–8.281)	0.324		
Pathologic M stage	272				
M0	268	Reference		Reference	
M1	4	4.077 (1.281–12.973)	0.017	1.074 (0.253–4.554)	0.923
Residual tumor	344				
R0	326	Reference			
R1 and R2	18	1.604 (0.812–3.169)	0.174		
Child–Pugh grade	240				
A	218	Reference			
B and C	22	1.643 (0.811–3.330)	0.168		
Fibrosis ishak score	214				
0&1/2	106	Reference			
3/4 and 5 and 6	108	0.740 (0.445–1.232)	0.247		
Adjacent hepatic tissue inflammation	236				
None	118	Reference			
Mild and Severe	118	1.194 (0.734–1.942)	0.475		
Albumin(g/dL)	299				
<3.5	69	Reference			
≥3.5	230	0.897 (0.549–1.464)	0.662		
SLC25A39	373				
Low	187	Reference		Reference	
High	186	2.002 (1.403–2.857)	< 0.001	1.638 (1.034–2.597)	0.036

**Table 3:** Relative mRNA expression of SLC25A39 in HCC cell lines (CCLE).

HCC	SLC25A39 mRNA Expression
HUH7	6.425089989874059
SNU739	6.447248568601776
SNU761	6.458775699080244
SNU475	6.5049381316496095
JHH6	6.536208354692263
HLF	6.596637034102074
JHH5	6.806452955763212
SNU182	6.84749588378871
KMCH1	6.8718436485093175
SNU387	6.885696373339395
HUH1	6.991521846075695
SNU886	7.012233435894742
JHH4	7.114262900594055
SNU423	7.144760196476028
JHH1	7.1851727751412495
HEP3B217	7.197511592107616
LI7	7.246598103057115
SNU398	7.290295375389055
SKHEP1	7.330379127580632
SNU878	7.4239142676731
PLCPRF5	7.488241842777295
JHH2	7.533329732305834
SNU449	7.59484709
JHH7	7.700439718141093

## Data Availability

The original contributions presented in this study are included in the article and Supplementary Materials. Further inquiries can be directed to the corresponding authors.

## Supplementary Materials

The following supporting information can be downloaded at: https://www.mdpi.com/article/10.3390/ijms27073098/s1.

## Abbreviations

The following abbreviations are used in this manuscript:

BP	biological processes
CC	cellular components
CCK-8	cell Counting Kit-8
DEGs	differentially expressed genes
DSS	disease-specific survival
GEO	gene Expression Omnibus
GO	gene ontology
GSEA	gene set enrichment analysis
GSH	glutathione
HCC	hepatocellular carcinoma
IHC	immunohistochemistry
KEGG	kyoto encyclopedia of genes and genomes
MF	molecular functions
OS	overall survival
PFS	progression-free survival
PPI	protein–protein interaction
qRT-PCR	quantitative reverse transcription polymerase chain reaction
RFS	relapse-free survival
ROC	receiver operating characteristic
SLC25A39	solute carrier family 25 member 39
ssGSEA	single-sample gene set enrichment analysis
STRING	search tool for the retrieval of interacting genes
TCGA	the cancer genome atlas
TGCT	testicular germ cell tumors
TIME	tumor immune microenvironment
