# Supplementary material for: SLC25A39 Upregulation Is Associated with DNA Methylation, Immune Cell Infiltration, and Poor Prognosis in Hepatocellular Carcinoma
Source: Int J Mol Sci. 2026 Mar 28;27(7):3098. doi: 10.3390/ijms27073098 (PMC13073517; doi:10.3390/ijms27073098)
Supplement: Supplementary file 1 [file ijms-27-03098-s001.zip › ijms-4212820-supplementary/supplementary figures.pdf]

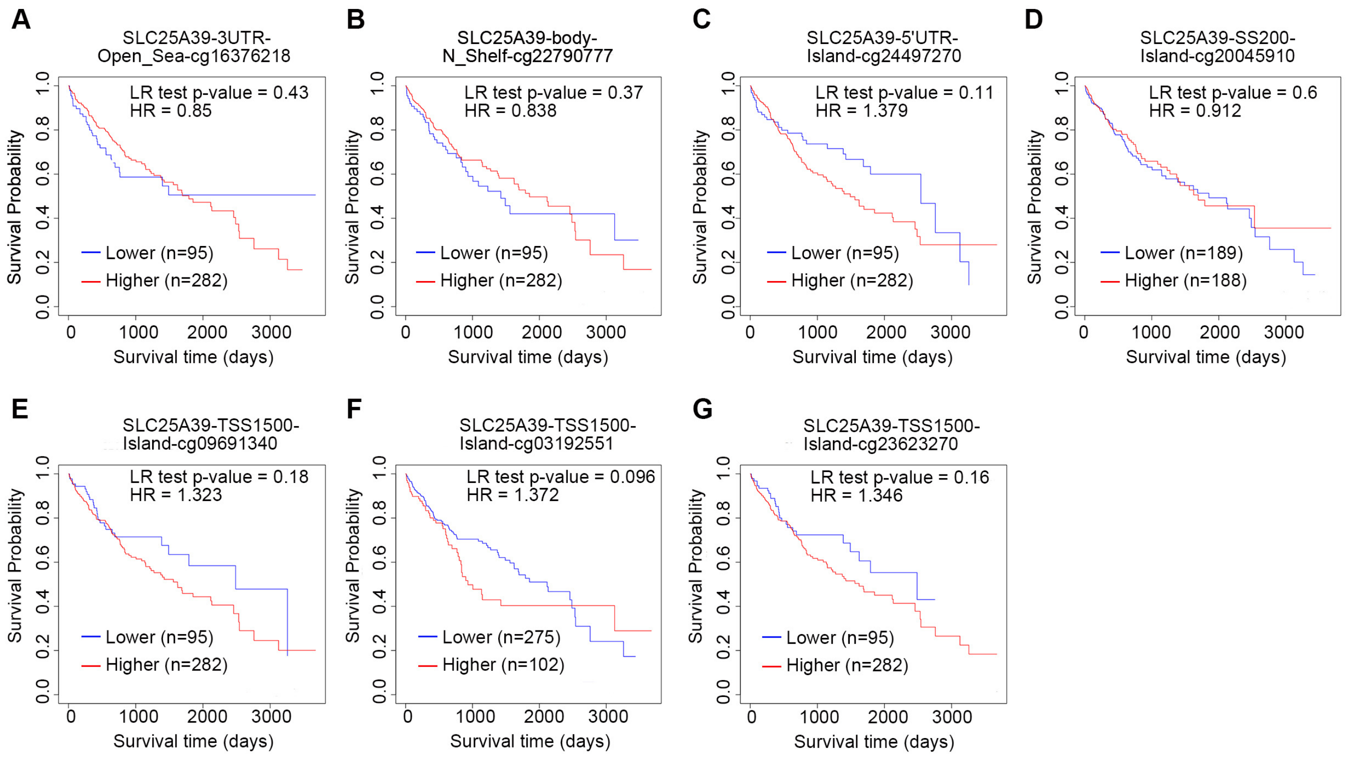

Figure S1. The association between SLC25A39 DNA methylation and prognosis in HCC.

A-G. Association between SLC25A39 methylation level and overall survival of HCC. Kaplan-Meier survival curve of SLC25A39 CpG methylated sites in (A) cg16376218, (B) cg22790777, (C) cg24497270, (D) cg20045910, (E) cg09691340, (F) cg03192551, (G) cg23623270.

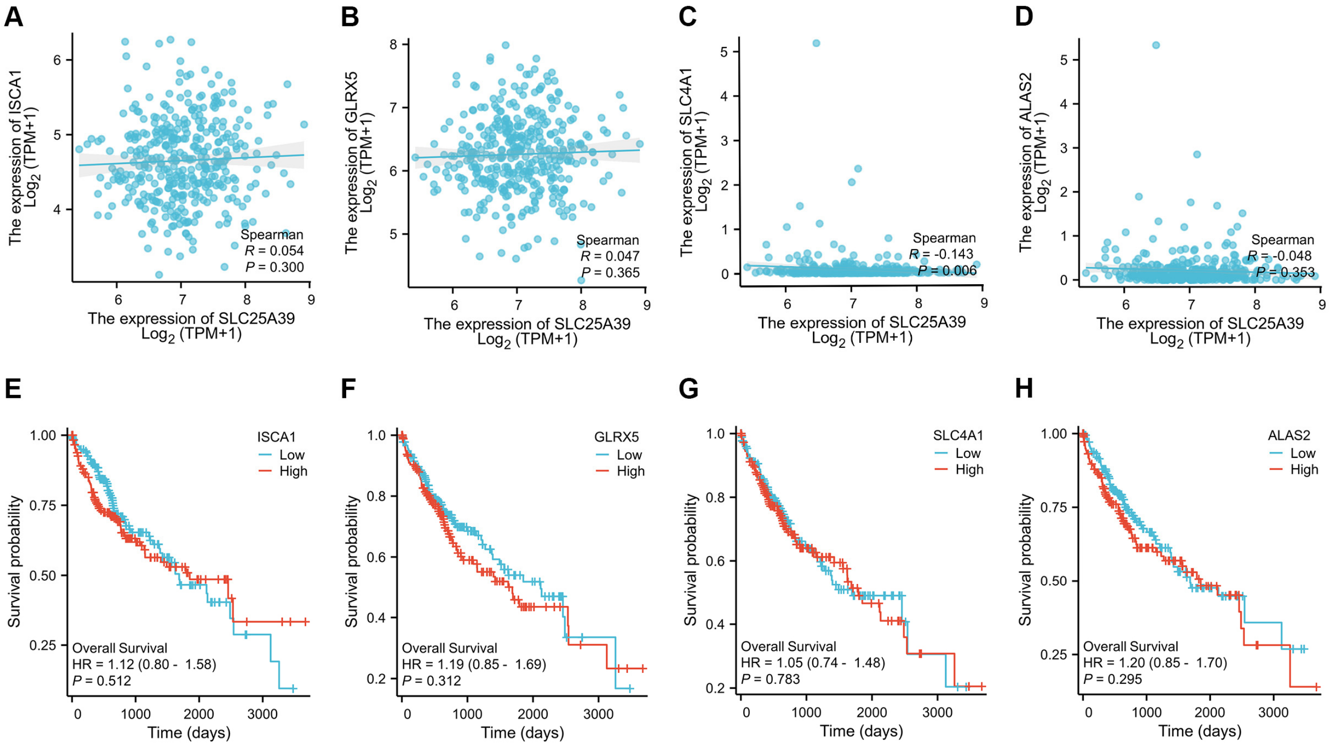

Figure S2. Correlation between SLC25A39 mRNA and SLC25A39-associated genes and their prognostic value in HCC from TCGA.

A-D. Correlation between SLC25A39 and (A) ISCA1, (B) GLRX5, (C) SLC4A1 and (D) ALAS2 from TCGA database.

E-F. Overall survival of (E) ISCA1, (F) GLRX5, (G) SLC4A1 and (H) ALAS2 in HCC from TCGA database.

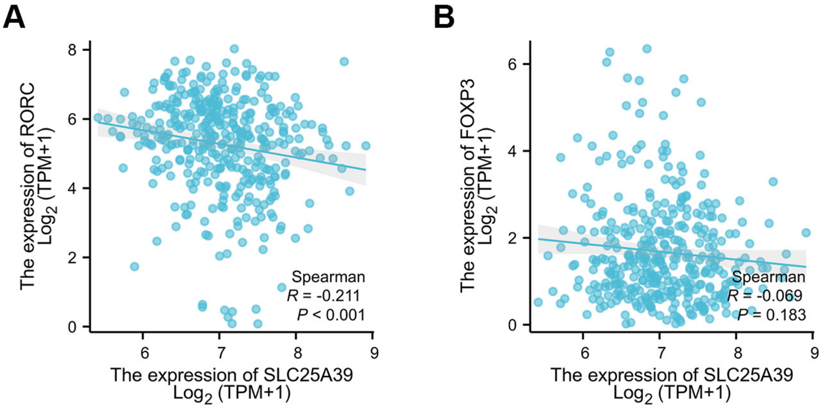

Figure S3. Correlation between SLC25A39 mRNA and markers of Th17 (RORC) and Treg (FOXP3).

A-B. Scatter plots depicting the correlations of SLC25A39 expression with (A) RORC and (B) FOXP3.
